# Spectrum of childhood nephrotic syndrome in Iran: A single center study

**DOI:** 10.4103/0971-4065.57103

**Published:** 2009-07

**Authors:** A. Safaei, S. Maleknejad

**Affiliations:** Department of Pediatrics, Guilan University of Medical Science, Rasht, IR, Iran

**Keywords:** Children, complication, corticosteroid, nephrotic syndrome, pathology

## Abstract

Nephrotic syndrome (NS) is a clinical entity characterized by massive loss of urinary protein (primarily albuminuria) leading to hypoproteinemia (hypoalbuminemia) and its result, edema. Hyperlipidemia, hypercholesterolemia, and increased lipiduria are usually associated. Although not commonly thought of as part of the syndrome, hypertension, hematuria, and azotemia may be present. This prospective cross-sectional study was performed on 44 children (with age of onset up to 14 years) with idiopathic nephrotic syndrome (INS) in our center during 2000–2007. The objectives were to study the clinical and biochemical parameters and the histopathological distribution of different subtypes of INS and drug-response pattern in these patients. The study group included 44 children. There were 29 (66%) boys and 15 girls (34%). The mean age was 4.87 ± 3.24 years. Facial edema was found in 42 (95%), microscopic hematuria in 10 (23%), gross hematuria in 2 (4.5%), and hypertension in 5 (11.2%) patients. In children who underwent biopsy, focal segmental glomerulosclerosis was the most common pathologic finding (41%). Other subtypes included minimal change disease in three (18%), membranoproliferative glomerulonephritis in one (5.8%), diffuse proliferative glomerulonephritis in two (11.6 %), membranous glomerulonephritis in one (5.8%), and diffuse mesangial proliferation in three (17.5%) cases. At the time of hospitalization peritonitis was present in five (11.4%), pneumonia and upper respiratory infection (sinusitis) in eight (18%), and cellulites in two (4.5%) patients. Twenty nine patients (66%) were steroid sensitive, 9 (20.5%) steroid resistant, and 6 (13.5%) steroid dependent. Among patients with steroid-sensitive NS, 37% were nonrelapsers, 38.8% frequent relapsers, and 26.4% were infrequent relapsers. Differences seems to exist between season of incidence, suitable response to treatment with corticosteroids, and pathologic findings of biopsy in our study and other studies from Iran and other countries.

## Introduction

Nephrotic syndrome (NS) is characterized by substantial loss of protein in the urine (primarily albuminuria), leading to hypoproteinemia (hypoalbuminemia) and its result, edema. Hyperlipidemia, hypercholesterolemia, and increased lipiduria are usually associated. Although not commonly thought of as part of the syndrome, hypertension, hematuria, and azotemia may also occur. NS is usually due to a glomerular disease and is currently categorized into primary and secondary forms. The primary NS (PNS) or idiopathic NS (INS) – both terms denote a similar vagueness as to cause – is not associated with any underlying disease. The syndrome manifests with varied clinical and pathologic states. The term secondary NS relates to ream of clinical diseases affecting the kidneys, such as anaphylactic purpura, systemic lupus erythematosus, diabetes mellitus, sickle cell disease, syphilis, and others. In the following sections, the majority of attention will be devoted to PNS/INS because of their relative frequency in children. The overall prevalence of NS in childhood is approximately 2–5 cases per 100,000 children. The cumulative prevalence rate is approximately 15.5 cases per 100,000.[1,2] Minimal change nephrotic syndrome (MCNS) is the most common form in children, and its prevalence is inversely proportional to the age (i.e., the younger the child, the more likely the histology will show minimal abnormalities on light microscopic evaluation of glomerular histology). Clear differences in racial predilection to NS and maybe even a more pronounced difference in the types of NS acquired within a geographic area exist, but the definitive data supporting these are lacking.[1,3–5] In children below eight years at onset, the ratio of males to females varies from 2:1–3:2 in various studies.[1,2,5] In older children, adolescents, and adults, the sex ratio is approximately equal.[4] The younger the child at onset (with the exception of the first few months of life), the greater the likelihood that the lesion is MCNS. Histologic variations exist within this category in which some patients demonstrate only fusion and smudging of the epithelial cell podocytes while others may demonstrate mild changes within the glomerular mesangium consisting of either proliferation or sclerosis. Since patients with MCNS have the highest rate of responsiveness to standard treatment and best long-term prognosis, the separation of MCNS from others is important.[6–8]

## Materials and Methods

This prospective cross-sectional study was performed on 44 children with INS from September 2000–2007 in 17 Shahrivar Hospital (with age at onset up to 14 years). All patients fulfilled the international study of kidney disease in children (ISKDC) criteria for the diagnosis of NS: nephrotic-range proteinuria (urinary spot protein/creatinine > 2.0), hypoalbuminemia (serum albumin < 2.5 g/dl), hyperlipidemia (serum cholesterol > 200 mg/dl), and edema.[1] The study parameters included age, sex, nationality, presenting symptoms, blood pressure, complete blood count, urinalysis and microscopy, 24-hour urinary protein excretion, creatinine clearance, serum electrolytes, serum urea and creatinine levels, serology and immunological studies, serological markers for hepatitis B and C, antibody against the human immunodeficiency virus (HIV), ultrasound, treatment provided, and outcome.

Following informed consent, kidney biopsy was performed in the following situations: (a) age at onset between zero and fourteen years, (b) no response to eight-week prednisolone therapy, (c) frequent relapser (FR), steroid-dependent (SD), and steroid nonresponder (SNR) categories, and before cyclosporine therapy, and (d) unusual clinical features (hypertension and micrscopic hematuria) and/or laboratory abnormalities (abnormal renal function and low C3 and C4 levels).[1,2,4] The biopsy specimens were evaluated histopathologically by light and immunofluorescence microscopy. An adequate biopsy was defined as the presence of at least ten glomeruli in the specimen on light microscopy. The response to treatment was classified according to the definitions from ISKDC: (a) steroid sensitive – complete resolution of proteinuria within eight weeks of prednisone therapy; (b) steroid resistance – failure to respond to eight consecutive weeks of treatment with prednisone at 2 mg/kg/day; (c) steroid dependence – recurrence of nephrosis when the dose of corticosteroids was reduced or within two months after the discontinuation of therapy; (d) frequent relapses – two or more episodes of nephrosis within six months of the initial response or four or more within a 12-month period (not related to changes in prednisone dose),[1,2] SD patients, FRs, and steroid-resistant patients candidates for alternative agents particularly, levamisol, cyclophosphamide, cyclosporine, mycofenolate mofetil, and tacrolimus.[1,9,10] Using a standardized data-sheet, we obtained data regarding age, sex, presenting features, laboratory findings, response to treatment, and biopsy results.

## Results

Forty four children with INS were analyzed during this period. There were 29 boys (66%) and 15 girls (34%) (M:F = 1.9/1). The mean age at onset of INS was 4.87 ± 3.24 years (age of onset up to 14 years). One of these patients had congenital NS. Fifteen patients were admitted to the hospital in spring (34.1%), others in winter (18.2%), summer (25%), and in fall (22.7%). The mean level of serum albumin was 1.75 ± 0.45, 24-hour urinary protein excretion was 3344.84 ± 2344.38 mg, serum cholesterol was 473 ± 160 mg/dL, and serum triglyceride was 335.4 ± 113.8 mg/dL. The most common presenting signs and symptoms were facial edema in 42 (95%), limb edema in 36 (82.2%), scrotal edema in 24 (54.5%), anasarca in 18 (41%), abdominal pain in 8 (18.2%), diarrhea in 4 (9%), ascitis in 28 (64%), pleurisy in 4 (9%), anorexia in 24 (54.5%), hypertension in 5 (11.2%), microscopic hematuria in 10 (23%), and gross hematuria in 2 patients (4.6%). All patients were seronegative for HBsAg and HIV. Evaluation for complications of the disease was done in all patients. Acute renal failure due to low serum albumin and overestimate diuretic consumption in other centers were seen in eight patients (18%). Acute renal failure recovered in seven patients. Biopsy was done in one patient because of persistent acute renal failure, and renal pathology was compatible with focal segmental glomerolusclerosis (FSGS). With regard to hospital admissions, peritonitis was present in five patients (11.4%), pneumonia and upper respiratory infection in eight (18%), and cellulities in two (4.5%). At follow-up, primary disease progress toward end stage renal disease (ESRD) occurred in four (9%) patients. Finally, renal transplantation was performed in two cases with FSGS, details regarding age and sex distribution of patients are presented in Table 1.

**Table 1:** Age and sex distribution of 44 patients with nephrotic syndrome

Age (years)	Boys	Girls	Total	Percent
<1	1	-	1	2.2
1–5	19	12	31	70
6–10	6	2	8	18.8
>10	3	1	4	9

Kidney biopsies were performed in 17 (38.4%) of 44 patients.

In children who underwent biopsy, FSGS was the most common histopathological subtype in 7 of 17 children (41%). Other subtypes included minimal change disease in three (18%), membranoproliferative glomerulonephriti (MPGN) in one (5.8%), diffuse proliferative glomerulonephritis in two (11.6%), membranous glomerulonephritis in one (5.8%), and diffuse mesangial proliferation (DMP) in three (17.5%). Among 44 NS children 29 (66%) were steroid sensitive, 9 (20.5 %) were steroid resistant, and 6 (13.5%) SD. Of patients with steroid-sensitive NS, 37% were nonrelapsers, 38.8% FRs, and 26.4% infrequent relapsers. Among those with steroid-resistant NS, seven had FSGS and two had DMP. In this study only one patient with FSGS died because of ESRD.

## Discussion

Patients with NS lose massive amounts of protein in the urine leading to hypoproteinemia and its result, edema. Hyperlipidemia, hypercholesterolemia, and increased lipiduria are also associated.[1,2] In this study, we analyzed all children with INS referred to 17 Shahrivar Hospital. In our case series of 44 pediatric patients, there were 29 boys (66%) and 15 girls (34%), M/F = 1.9/1; in other studies this gender ratio from 1.6 to 2.76/1.[6–8,11,12]

In our report, 31 patients were in the age range 1–5 years (71%). Only one patient had congenital NS at 45 days. The mean age of patients at onset of INS was 4.87 ± 3.24 years. In the study by Kumar *et al*, in India, the mean age of patients at onset of NS was 7.9 ± 5.1 years,[11] and in other studies of New Zealand and Saudi Arabia showed that the mean age patients was 5.4 ± 3.9 and 4.3 ± 3.1 years, respectively.[13,14] In another Iranian study, 67% of 502 patients were in the age range of 1–5 years, whereas 62.5% patients were in the range of 2–8 years in another.[12] Our findings are relatively similar to results obtained in two centers from Iran. In this study, 15 patients were admitted to the hospital in spring season (34.1%), other patients in winter (18.2%), summer (25%), and fall (22.7%), but in the report of Sorkhi *et al*, 38% of cases were admitted in winter season.[12] In our study, presenting signs and symptoms in comparison with other studies were different, for example frequency of microscopic hematuria and hypertension in Indian children was 41 and 26.8%, respectively.[11,14]

Of the biopsied children, FSGS was the most common histopathological subtype (41%). Biopsies in Turkish children showed that pathologic findings compatible to mesangial proliferate glomerulonephritis in 49% cases,[6,7] whereas FSGS was the most common histopathological subtype in Indian children (38%). In the study by Madani *et al*, minimal change disease was the most common histopathological subtype (34.4%).[8] This study in comparison with other studies from other countries and centers showed variable histological patterns, although at the moment it seems that minimal change disease is the most common variation of NS in children. These differences may be related to racial, genetic, and environmental characteristics.[3,6,11,13,15] Out of the 44 children, 29 (66%) were steroid sensitive, 9 (20.5%) steroid resistant, and 6 (13.5%) were SD. Among those with steroid-resistant NS, seven patients had FSGS and two had DMP. Other studies have shown variable corticosteroid sensitivity of 76–87%.[6,8,10,16,17–19] In our study, one patient had congenital NS and another patient died of complications of ESRD.[20]

## Conclusion

The seasons at clinical presentation, hence the incidence timing; histopathological findings of biopsies; and suitable response to treatment with corticosteroids are different from other studies conducted in Iran and other countries.
